# Therapeutic effect of fenofibrate for non-alcoholic steatohepatitis in mouse models is dependent on regime design

**DOI:** 10.3389/fphar.2023.1190458

**Published:** 2023-05-12

**Authors:** Xinxue Wang, Jia Luo, Zhuoheng Lu, Shenzhe Fang, Mengxia Sun, Wenjing Luo, Jianwei Shen, Aiming Liu, Hua Ye

**Affiliations:** ^1^ Department of Gastroenterology, The Affiliated Lihuili Hospital of Ningbo University, Ningbo, China; ^2^ Zhejiang Key Laboratory of Pathophysiology, Department of Pharmacology, Health Science Center, Ningbo University, Ningbo, China

**Keywords:** non-alcoholic steatohepatitis, fenofibrate, peroxisome proliferator activated receptor α, methionine choline-deficient diet, choline-deficient L-amino acid-defined high-fat diet, therapeutic action

## Abstract

**Background:** Non-alcoholic fatty liver disease (NAFLD) is the leading cause of chronic liver diseases. In most cases, NAFLD progresses from benign steatosis to steatohepatitis (NASH), and then to cirrhosis. No treatment is currently approved for NAFLD/NASH in the clinic. Fenofibrate (FENO) has been clinically used to treat dyslipidemia for more than a half century, but its effects on NASH are not established. FENO’s half-life is quite different between rodent and human. The aim of this study was to investigate the potential of pharmacokinetic-based FENO regime for NASH treatment and the underlying mechanisms.

**Methods:** Two typical mouse NASH models, methionine-choline deficient (MCD) diet-fed mice and choline-deficient, L-amino acid-defined, high-fat diet (CDAHFD)-fed mice, were used. MCD model was designed as therapeutic evaluation in experiment 1 and CDAHFD model was designed as preventive in experiment 2. Three doses of FENO (5, 25, 125 mg/kg), two times a day (BID), were administered to the above models. Serum markers of liver injury, cholestasis, and the histology of liver tissues were investigated. Normal mice were used as a model in experiment 3 for toxicity evaluation, Quantitative-PCR and Western Blot assays were used to investigate the inflammatory responses, bile acid synthesis as well as lipid catabolism.

**Results:** Mice on the MCD and CDAHFD diets developed steatohepatitis as expected. Treatment with FENO (25 mg/kg·BID) significantly decreased hepatic steatosis, inflammation and fibrosis in both therapeutic and preventive models. In the MCD model, the therapeutic action of FENO (25 mg/kg·BID) and 125 mg/kg·BID on histopathology and the expression of inflammatory cytokines were comparable. In reducing macrophage infiltration and bile acid load, FENO (25 mg/kg·BID) was superior to 125 mg/kg·BID. In all the aspects mentioned above, FENO (25 mg/kg·BID) was the best among the 3 doses in the CDAHFD model. In a third experiment, the effects of FENO (25 mg/kg·BID) and 125 mg/kg·BID on lipid catabolism were comparable, but 125 mg/kg·BID increased the expression of inflammatory factors and bile acid load. In both models, FENO (5 mg/kg·BID) showed little effect in hepatic steatosis and inflammation, neither the adverse effects. FENO (125 mg/kg·BID) aggravated liver inflammation, increased bile acid synthesis, and promoted the potential of liver proliferation. In toxicity risk assay, FENO (25 mg/kg·BID) treatment showed low potential to trigger bile acid synthesis, inflammation and hepatocyte proliferation.

**Conclusion:** A new regime, FENO (25 mg/kg·BID) is potentially a therapeutic strategy for the NASH treatment. Translational medicine is warranted to prove its effectiveness in the clinic.

## 1 Introduction

Non-alcoholic fatty liver disease (NAFLD) is a chronic metabolic disease with complex etiological and pathophysiological backgrounds, characterized by prominent hepatocyte steatosis. The reported prevalence of NAFLD is 25%–30%, putting global public health at high risk (Younossi et al., 2018). In China, the biggest developing country, 7%–30% of patients with NAFLD proceed to non-alcoholic steatohepatitis (NASH), and some of them advance to cirrhosis and liver cancer within 5–10 years (Younossi et al., 2016). Animal and clinical studies on NASH demonstrated therapeutic outcomes, suggesting NASH was reversible and curable. However, no effective therapy is approved for NASH treatment in the clinic presently. Thus, it is important to find new strategies to deal with NASH.

Peroxisome proliferator activated receptor alpha (PPARα) is a nuclear receptor that binds fatty acids and their derivatives. It regulates the expression of genes involved in fatty acid metabolism in the liver (Liss and Finck, 2017). Clinical fibrate agents are PPARα agonists which have been drugs of choice to treat dyslipidemia for more than 50 years. Of them, fenofibrate (FENO) is the most popularly used due to its least toxicity risk (Pahan, 2006; Arai et al., 2018). In recent years, FENO was found to be effective in treating metabolic diseases, such as metabolic syndrome, cholestatic liver diseases, diabetic mellitus and acute liver injury (Li et al., 2009; Ghonem et al., 2015; Gomaraschi et al., 2015; Harmer et al., 2015; Zhang et al., 2018). A typical daily dose of 160 mg/day FENO increased high density lipid-cholesterol and alleviated metabolic syndrome (Gomaraschi et al., 2015). FENO (150–200 mg/day) decreased biochemical markers and cholestatic symptoms in chronic cholestasis (Ghonem and Boyer, 2013; Ghonem et al., 2015). 200 mg/day of FENO was found effective on blood glucose in patients with type 2 diabetes (Harmer et al., 2015). FENO (100 mg/kg·day) reduced acetaminophen-induced acute liver injury by inhibiting inflammation (Yamazaki et al., 2002; Zhang et al., 2018). These studies demonstrated the efficacy of FENO at regular dose levels in the treatment of metabolic diseases.

Some groups reported their efforts trying FENO therapy for NAFLD. Studies showed FENO improved liver function, liver steatosis, and inflammatory response in NASH patients and mice. In high-fat diet (HFD)-fed mice, FENO (100 mg/kg day) decreased serum alanine aminotransferase/aspartate aminotransferase (ALT/AST) levels and the amount of liver fat (Yoo et al., 2021). In methionine-choline deficient (MCD)-fed mice, FENO (100 mg/kg·day) reduced serum ALT levels and improved liver morphology (Chanda et al., 2009). Combination of FENO (10 mg/kg·day) and Acetyl-CoA carboxylase ameliorated hypertriglyceridemia and stimulated fatty acid oxidation in high fat, high cholesterol diet-fed mice (Goedeke et al., 2018). In clinical trials, FENO (200 mg/day) reduced blood glucose, serum triglycerides (TG) and alkaline phosphatase (ALP) without side effects (Fernández-Miranda et al., 2008). The same dose regime decreased a various lipid biomarkers in obese-NAFLD patients (Fabbrini et al., 2010). FENO (300 mg/day) significantly improved hepatic fibrosis, inflammation and liver stiffness in NASH patients (El-Haggar and Mostafa, 2015). These results demonstrated the therapeutic effects of FENO in treating NAFLD/NASH.

FENO therapy, however, was also found to worsen hepatic steatosis and increased the inflammatory responses in NASH in several studies. FENO (100 mg/kg·day) demonstrated only minimal improvements in hepatic steatosis in NASH mice (Jain et al., 2018). At a daily dosage of 400 mg/kg, FENO treatment caused accumulation of fat in the liver in a mouse model (Le et al., 2014). In the clinic, FENO (200 mg/day) lowered serum TG level but increased total liver fat content, demonstrating that FENO had complicated effects on human hepatic lipid metabolism (Oscarsson et al., 2018). FENO (200 mg/day) did not significantly improve liver histology in NAFLD patients (Fernández-Miranda et al., 2008). Combining the above, present data about FENO actions in NASH treatment were still controversial.

Controversial conclusion about FENO therapy for NASH might be attributed to protocol design. In this study, three doses of FENO (5, 25, and 125 mg/kg) were administered intragastrically, two times a day (BID, regime designed based upon the half-life in rodent), to evaluate their actions on methionine-choline deficient (MCD) and choline-deficient, L-amino acid-defined, high-fat diet (CDAHFD)-induced NASH models. The MCD model was therapeutic in experiment 1, and the CDAHFD model was preventive treatment in experiment 2. Meanwhile, the toxicity risk of FENO treatment were evaluated in experiment 3. Conclusion were made based on combination of pathological phenotype, anti-inflammation, biochemistry and toxicity risk.

## 2 Materials and methods

### 2.1 Drugs and reagents

FENO was obtained from Shanghai Aladdin Biochemical Technology Co., Ltd. Sodium carboxymethyl cellulose (CMC-Ma) was obtained from Sangon Biological Co., Ltd. The detection kits for total cholesterol (TC), triglyceride (TG), alanine aminotransferase (ALT), aspartate aminotransferase (AST), alkaline phosphatase (ALP), and total bile acid (TBA) were acquired from Ningbo Meikang Biotechnology Co., Ltd. Trizol was purchased from Ambion Invitrogen in the United States. Hematoxylin-eosin (HE) and Masson-Trichrome (MT) staining kits were purchased from Shanghai Sobo Biotechnology Co., Ltd. Reverse transcription and quantitative PCR (Q-PCR) kits were produced by Beijing Kangwei Century Biotechnology Co., Ltd. F4/80 antibody was purchased from Abcam (MA, United States). BCA Protein Assay Kit was purchased from Beyotime Biotechnology Co., Ltd. Antibodies against total protein 38 (t-p38), phosphorylated protein-38 (p-p38), total extracellular regulated protein kinases 1/2 (t-ERK1/2) and phospho-extracellular regulated protein kinases 1/2 (p-ERK1/2) were obtained from ABclonal Technology Co., Ltd. Total JNK (t-JNK) and phospho-JNK (p-JNK) were bought from Cell Signaling Technology Co. Ltd. Total extracellular regulated protein kinases 5 (t-ERK5) and phospho-extracellular regulated protein kinases 5 (p-ERK5) were purchased from Santa Cruz Biotechnology Co., Ltd. The goat anti-mouse IgG and goat anti-rabbit IgG were bought from Proteintech Co., Ltd.

MCD diet (SYSE Biotechnology Co. Ltd., Jiangsu, China) contains (per 1,000 g): amino acid premix (methionine free) 175.7 g, sucrose 431.9 g, dextrin 50 g, corn starch 150.0 g, corn oil 100.0 g, cellulose 30.0 g, mineral mix 52.4 g. CDAHFD diet (SYSE Biotechnology Co. Ltd., Jiangsu, China) contains (per 1,000 g) amino acid premix 349.2 g, methionine 1.6 g, sucrose 345.6 g, corn starch 154.2 g, soybean oil 50.0 g, cellulose 100.0 g, mineral mix 20.0 g, vitamin mix 20.0 g.

### 2.2 Animal and experimental protocols

Seventy male C57BL/6 mice (SCXK (Shanghai) 2017-0005) were obtained from Shanghai Slack Laboratory Animal Co., Ltd., which were 7 weeks old and weighed 23–25 g. Mice were allowed access to normal chow and water *ad libitum* for 1 week. After 1 week of acclimatization, the mice were randomly assigned into three experiment plans.

In the first experiment, the mice were divided into five groups: MCD-CON group; MCD group; MCD-FENO (5, 25, 125 mg/kg·BID; MF-5, 25, 125) groups (n = 5). Mice were fed the MCD for 5 weeks to establish a NASH model (Jain et al., 2018; Deng et al., 2020). After 5 weeks of MCD feeding, FENO (5, 25 and 125 mg/kg·BID) was administered by oral gavage for treatment therapy for 3 consecutive weeks (Dai et al., 2017).

In the second experiment, the mice were divided into five groups: CDAHFD-CON group; CDAHFD group; CDAHFD-FENO (5, 25, 125 mg/kg·BID; CF-5, 25, 125) groups (n = 5). CDAHFD diet was used to treat the diet and FENO (5, 25 and 125 mg/kg·BID) was orally gavaged concurrently for preventative therapy for 3 consecutive weeks. The body weight of the mice was measured every week during the experiment.

In the third experiment, 20 mice were divided into four groups: CON group; F5, F25, F125 groups. The CON group was treated with normal diet. F5, 25, 125 groups were treated with 5, 25, 125 mg/kg FENO, twice a day by oral gavage for 5 consecutive days. The aim of experiment 3 was to evaluate the toxicity risk of FENO. The expression of inflammatory factors, lipolysis factors and bile acid loading proteins were examined. FENO was administered two time everyday, at 8 a.m. and 8 p.m., with a 12-h interval between doses in three experiments. FENO was dissolved in 1% CMC-Na for administration.

All CON groups in 3 experiments (MCD-CON, CDAHFD-CON and CON group) were treated with 1% CMC-Na by oral gavage.

The body weight of mice was measured weekly. Body weight and liver wet weight were measured when mice were sacrificed. The ratio of liver wet weight to body weight (LW/BW) was calculated as the liver index. Blood samples were collected after the mice were sacrificed. They were centrifuged at 3,600 rpm for 15 min at 4°C to get the plasma. The supernatant was collected and stored at −80°C. Half of the biggest liver lobe was excised, fixed in 10% formalin, dried, and embedded in paraffin. The leftover of liver tissues was flesh frozen and kept at −80°C pending analysis.

### 2.3 Biochemical analysis

Biomarkers TC, TG, ALT, AST, TBA and ALP in serum were assayed using the Multiskan GO (Thermo, United States). The analyses were carried out following the manufacturers’ procedures described in the kits.

### 2.4 Measurement of fat level in the liver

20 mg of liver tissues were mixed with 180 μL of saline in a tissue homogenizer for 30 s at 4°C. Liver tissues were treated at 4°C at 13,000 rpm for 20 min, then the supernatant was transferred to an Eppendorf tube devoid of enzymes. Liver TC and TG were assayed using the Multiskan GO (Thermo, United States). The analyses were carried out following the same protocols as the above serum biomarkers.

### 2.5 Histopathological assessment

Fixed liver tissues in the above experiments were dehydrated in a serial concentration of alcohol and xylene, followed by paraffin embedding. Four-micrometer serial sections were cut and stained with hematoxylin-eosin (HE) and Masson-Trichrome (MT). Histopathological examination was performed using an Olympus BX51light microscope (LEICA, Wetzlar, GER). The non-alcoholic fatty liver activity score (NAS) was assessed based on HE staining (Kleiner et al., 2005). The score was defined as a sum of the scores for steatosis (0-3), lobular inflammation (0-3), and ballooning (0-2), ranging from 0 to 8.

### 2.6 Immunohistochemical staining

Paraffin-embedded liver sections (4 μm thickness) were deparaffinized in xylene and rehydrated in a gradient of ethanol to distilled water. Sections were incubated in 3% H_2_O_2_ for 5–10 min at room temperature before being washed with distilled water. After 5 min in phosphate buffer saline (PBS), the sections were blocked in 5%–10% PBS-diluted normal goat serum. Finally, the sections were incubated at 22°C for 10 min. Then, the primary antibody (F4/80) was added for 15 min of incubation at room temperature. A secondary antibody was added for 10–30 min incubation at 37°C and rinsed with PBS for 5 min × 3 times. Chromogenic agent 1, 2-diacetylbenzene (DAB) was used for development and visualization. Photomicrographs of liver tissue sections were taken using an Olympus BX51light microscope and positively immunostained area was quantified with Image J software.

### 2.7 Quantitative PCR analysis

The total RNA from mice hepatic tissues (20 mg) homogenized in Trizol reagent were determined by Multiskan Go (Thermo Scientific, Waltham, MA, United States). The reverse transcription system (20 μL) included the following items: 5× Reaction buffer 4 μL, total RNA 1 μg, Oligo dT18 1 μL, Random primer 1 μL, 10 mM dNTPs Mix 2 μL, 200 U RevertAid M-MuLV RT 1 μL and 20 U Ribolock RNase inhibitor 1 μL. The cDNA synthesized was stored at 20°C and subjected to analysis within 7 days. The primer sequences were extracted from https://pga.mgh.harvard.edu/primerbank. Each 10 μL PCR system contained 1 μL total cDNA, Light Cycle 480 SYBR Green I Master Mix (FastStart Taq DNA polymerase, reaction buffer, dNTP mix, SYBR Green I dye, and MgCl2) 5 μL, forward primer 0.2 μL, reverse primer 0.2 μL, nuclease-free water 3.6 μL. Amplification was performed using a reaction cycle at 95°C 10°s, 55°C 10 s and then 72°C 15 s. The fluorescence signal was detected at the end of each cycle. 18S rRNA was used as an internal control, and 2^-△△Ct^ was used to calculate the expression of the target genes. Primers for *Ccl2*, *Cxcl2*, *Tnf-α*, *Il-10*, *Cyp4α10*, *Acot1* and *Cpt1* were shown in Supplementary Table S1.

### 2.8 Western Blot analysis

The fresh liver tissue was lysed by RIPA and PMSF at a ratio of 100:1. The insoluble material was removed by centrifugation (12,000 rpm) for 20 min at 4°C. The protein concentration in the supernatant was determined by the BCA protein assay kit and then adjusted to 8 mg/mL. Then, 5 μL of each sample was loaded using SDS-polyacrylamide gels and transferred to PVDF membranes after electrophoresis. PVDF membranes were blocked with 5% skimmed milk for 3.5 h at room temperature. Next, membranes were incubated with primary antibodies overnight at 4°C. Finally, the blotted PVDF membranes were incubated with secondary antibodies for 2 h at room temperature after being washed 3 times using TBST. The washed membranes were exposed to ECL substrates (Advansta, Menlo Park, CA, United States) and the signals were detected by Tanon 4200SF (Tanon, Shanghai, China). The bands were quantified using Image J software.

### 2.9 Statistical analysis

The assignment of mice to different groups was randomized. All the data were presented as mean ± SD. The statistical analysis was performed using the software SPSS Statistics, version 25 (IBM, Beijing, China). Differences among multiple groups were tested using *one-way ANOVA* followed by *Dunnett’s post hoc* comparisons. Comparison between normal and model groups was tested by *Student’s t-test*. The difference was considered significant when *p* < 0.05 and was marked with an asterisk in the graphs accordingly.

## 3 Results

### 3.1 FENO 25 mg/kg·BID inhibited the inflammatory injury in two NASH models

**FIGURE 1:**
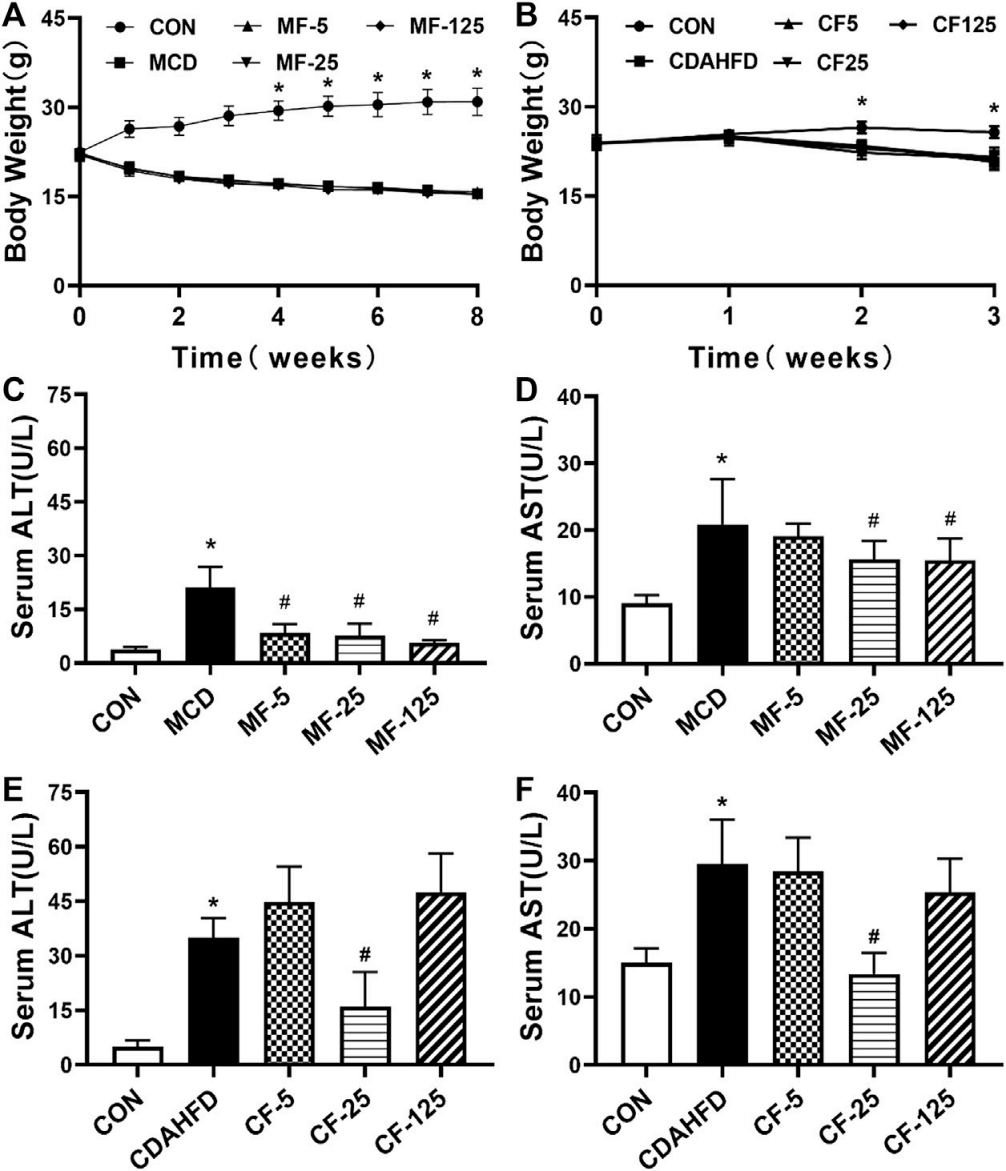
Effects of FENO on body weight and liver function index in serum in the MCD and CDAHFD-induced NASH mice. **(A, B)** The evolution of body weight. **(C–F)** The liver function index (ALT and AST) in serum. Data are presented as mean ± SD values. n = 5 mice per group. Significance was determined using Student’s *t*-test (*p < 0.05 versus MCD and CDAHFD-CON group) or Dunnett’s test (^#^ *p*< 0.05 versus MCD and CDAHFD group).

In the therapeutic MCD model, compared with MCD-CON group, a substantial decrease of body weight in the MCD group followed the MCD diet for 5 weeks (*p*< 0.001). The body weight decreased in MCD-FENO groups and there was no significant difference among the 3 MCD-FENO groups (Figure 1A). In the CDAHFD model, as a preventive treatment model, the body weight slightly decreased in both CDAHFD and CDAHFD-FENO groups compared with CDAHFD-CON group. Similarly, there was no statistical difference between CDAHFD and the 3 CDAHFD-FENO groups (Figure 1B). These data indicated that FENO treatment did not significantly alter the body weight in both NASH models.

As key indicators of liver injury, serum ALT and AST levels were 5 and 2 folds higher in the MCD group in comparison with the MCD-CON group (*p* = 0.002 and *p* = 0.018, respectively). In group MF-25, the above indicators were inhibited by 63% and 25% respectively (*p* < 0.001 and *p* = 0.044). In the MF-5 and MF-125 groups, serum ALT was reduced by 60% and 73% respectively (*p* < 0.001, Figures 1C, D). Serum AST was only reduced by 9% and 26% in the MF-5 and MF-125 groups.

In the preventive CDAHFD model, compared to the CDAHFD-CON group, serum ALT and AST increased by 7- and 2-fold ((*p* < 0.001) in the CDAHFD group. In the group CF-25, compared to theh CDAHFD group, serum ALT and AST concentrations were decreased by 54% and 55% (*p* < 0.001). Compared to the CDAHFD group, serum ALT in the CF-5 group and the CF-125 group was increased by 22% and 26%, respectively. In the CF-5 and CF-125 groups, serum AST was only decreased by 3% and 14% (Figures 1E, F). These results showed that in the 3 CDAHFD-FENO groups, FENO (25 mg/kg·BID) reduced the inflammatory injury with the best effect.

### 3.2 Pathological responses by FENO 25 mg/kg·BID were the best

**FIGURE 2:**
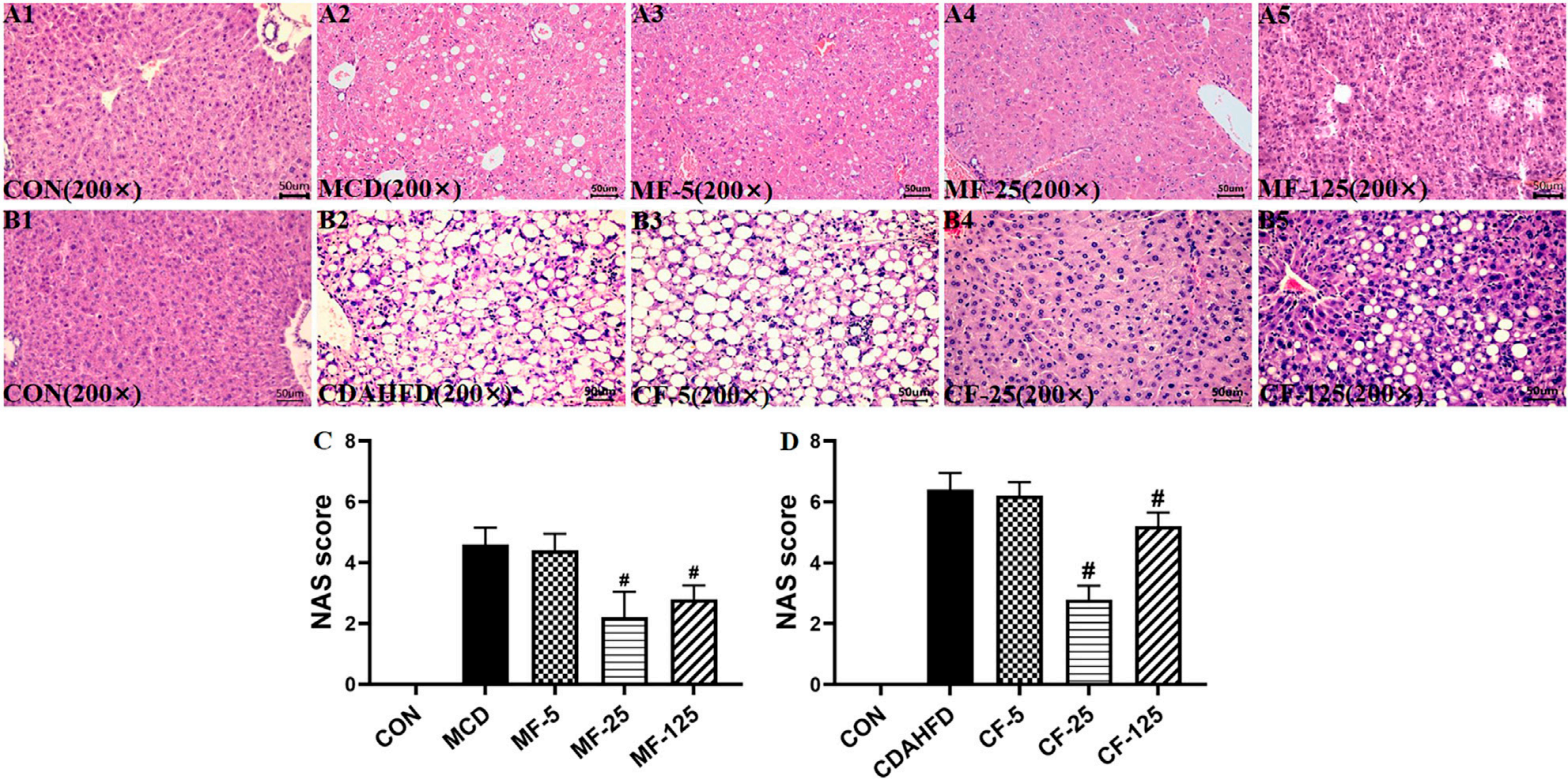
FENO 25 mg/kg·BID improved pathological responses in HE staining. Bar = 50 μm **(A1)**, **(B1)** HE staining of liver tissues in the MCD and CDAHFD-CON groups. **(A2)**, **(B2)** HE staining of liver tissue in the MCD and CDAHFD groups. **(A3)**, **(B3)** HE staining of liver tissues in the MF-5 and CF-5 groups, respectively. **(A4)**, **(B4)** HE staining of liver tissue in the MF-25 and CF-25 groups, respectively. **(A5)**, **(B5)** HE staining of liver tissues in the MF-125 and CF-125 groups, respectively. **(C)** NAS score in the MCD model. **(D)** NAS score in the CDAHFD model. (*n* = 5, *p < 0.05, compared with CON group; ^#^ *p*< 0.05, compared with MCD or CDAHFD group).

H&E staining showed that the liver of the CON groups in the two models appeared normal. The liver showed bright red color and the structure of the hepatic lobule was complete. The hepatocytes were arranged in a radial pattern around the central vein and the liver was absent of steatosis, inflammation and necrosis. In contrast, hepatic steatosis and inflammatory cell infiltration were quite evident in the MCD and CDAHFD groups. After FENO (25 mg/kg·BID) treatment, the lipid accumulation of the liver was significantly reduced in two models (Figure 2A1–A5, Figure 2B1-B5). Compared with MCD group, the NAS score decreased by 4%, 52% (*p* < 0.001) and 39% (*p* = 0.001) in the MF-5, 25, 125 groups, respectively. Compared with CDAHFD group, the NAS score decreased by 3%, 56% (*p* < 0.001) and 19% (*p* = 0.003) in the CF-5, 25, 125 groups, respectively. In two NASH models, the decrease in NAS scores was statistically significant at FENO 25 and 125 mg/kg·BID (Figures 2C, D).

**FIGURE 3:**
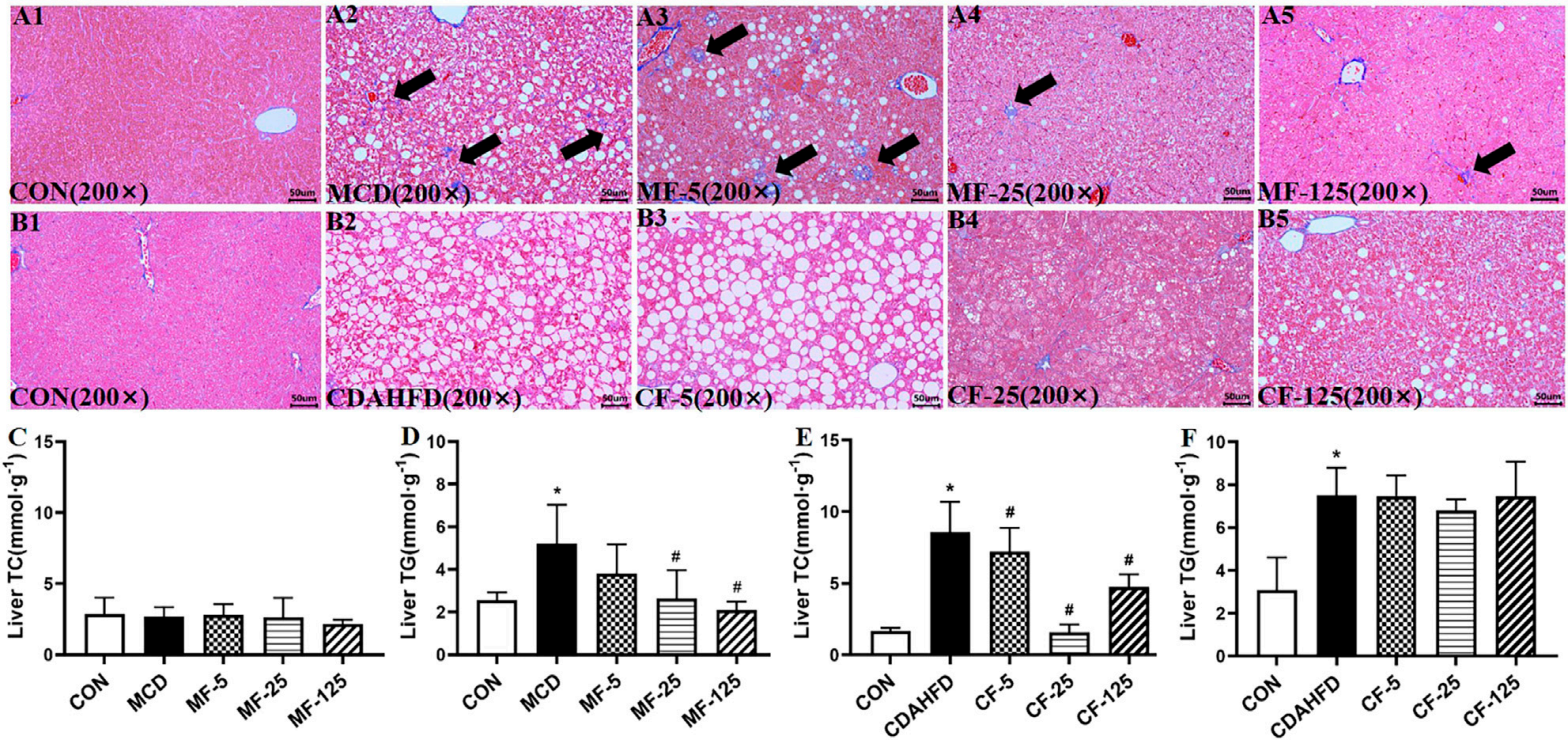
FENO 25 mg/kg·BID inhibited fibrosis in MT staining. Bar = 50 μm **(A1)**, **(B1)** Masson-Trichrome (MT) staining of liver tissues in the MCD and CDAHFD-CON groups. **(A2)**, **(B2)** MT staining of liver tissues in the MCD and CDAHFD groups. **(A3)**, **(B3)** MT staining of liver tissues in the MF-5 and CF-5 groups, respectively. **(A4)**, **(B4)** MT staining of liver tissues in the MF-25 and CF-25 groups, respectively. **(A5)**, **(B5)** MT staining of liver tissues in the MF-125 and CF-125 groups, respectively. **(C, D)** TC and TG in liver of the MCD model. **(E, F)** TC and TG in liver of the CDAHFD model. (*n* = 5, *p < 0.05, compared with CON group; ^#^ *p*< 0.05, compared with MCD or CDAHFD group).

MT staining revealed significant fibrotic changes in the liver tissue of the MCD model. Hepatic collagen deposition was seen in the MCD and MF-5 groups, indicating the formation of liver fibrosis. Hepatic collagen deposition was reduced in MF-25 and MF-125 groups (Figures 3A1–A5). There was no substantial difference in liver fibrosis between CDAHFD groups, probably due to the short treatment time of CDAHFD (Figures 3B1–B5).

Biochemically, the liver TG level in the MCD group increased by 51% compared with the MCD-CON group (*p* = 0.035), suggesting lipid deposition in the liver. After FENO treatment, the liver TG levels of the MF-5, MF-25, and MF-125 groups decreased by 27%, 50% (*p* = 0.024), and 60% (0.007), respectively. For the liver TC, there was no significant difference amount the 5 groups (Figures 3C, D). In the preventive CDAHFD model, the liver TC and TG concentrations in the CDAHFD group increased by 80% and 59% compared with the CDAHFD-CON group (*p* < 0.001). But the liver TC was reduced by 82% in the CF-25 group (*p* = 0.019), which was the strongest among the 3 dose levels. Similarly, there were no significant changes in liver TC levels between the CDAHFD, CF-5, CF-25, and CF-125 groups (Figures 3E, F).

### 3.3 Anti-inflammation of FENO 25 mg/kg·BID was strongest in two NASH models

**FIGURE 4:**
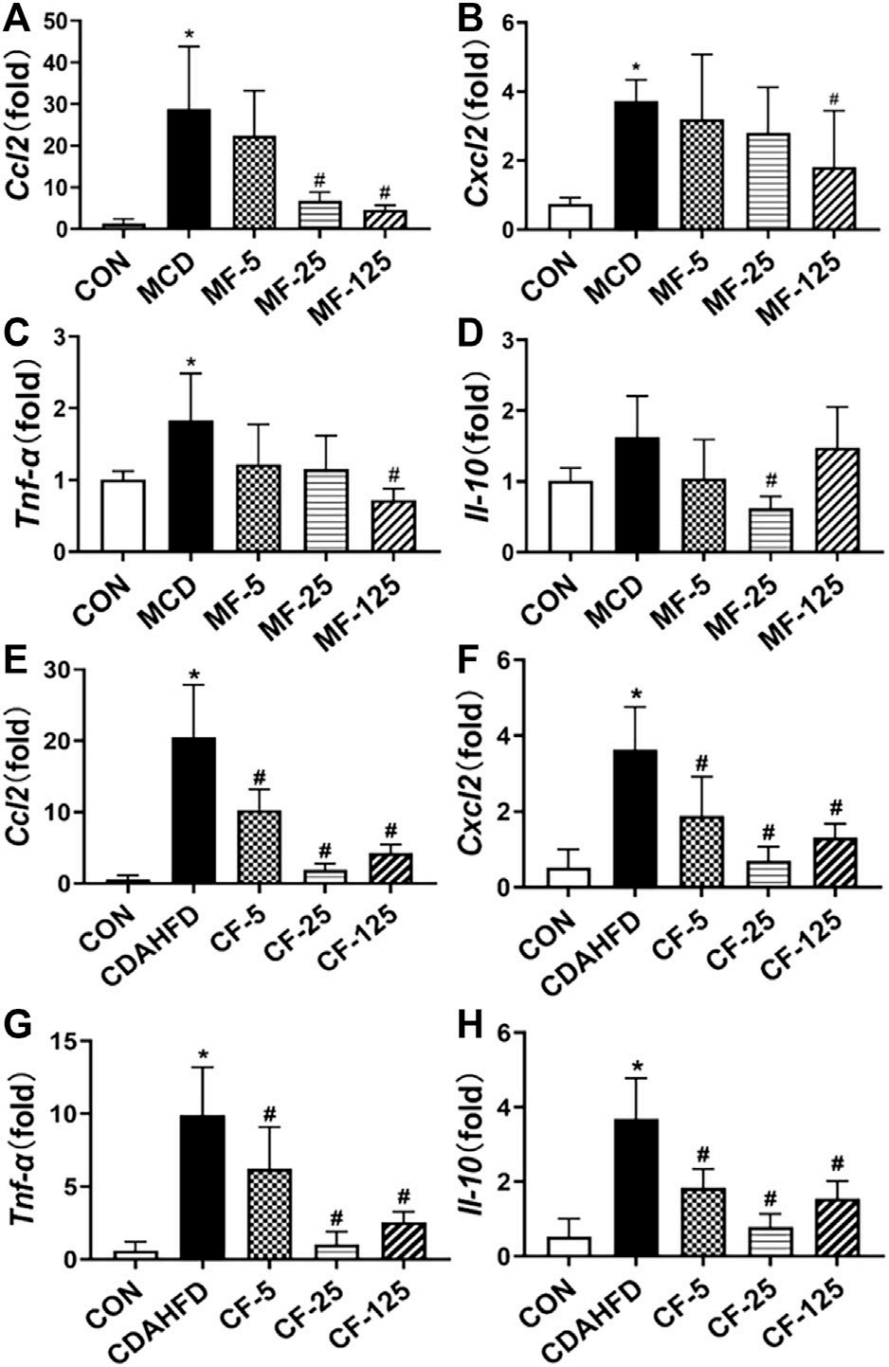
Effects of FENO on the transcription of genes involved inflammation. **(A, E)** *Ccl2* mRNA level in two mouse lines. **(B, F)** *Cxcl2* mRNA level in two mouse lines. **(C, G)** *Tnf-α* mRNA level in two mouse lines. **(D, H)** *Il-10* mRNA level in two mouse lines. The mRNA levels were measured by quantitative PCR and normalized by *18S rRNA*. The data are expressed as mean ± SD (*n* = 5, *p < 0.05, compared with CON group; ^#^ *p*< 0.05, compared with MCD or CDAHFD group).

In the therapeutic MCD model, the inflammatory chemokine *Ccl2* and *Cxcl2* levels in the MCD group increased 22-fold and 5-fold, respectively (*p*< 0.001 and *p* = 0.017), when compared to the MCD-CON group. The levels of *Ccl2* mRNA in MF-25 and MF-125 groups decreased by 76.5% and 83.5%, respectively (*p* = 0.015 and *p* = 0.009). *Cxcl2* level was decreased by 14%, 25%, and 51% (*p* = 0.035), respectively, in the MF5, MF25, and MF125 groups (Figures 4A, B). In addition, the mRNA levels of pro-inflammatory cytokine *Tnf-α* and protective inflammatory factor *Il-10* in the MCD group’s liver tissues were twice as high as those in the MCD-CON group. *Tnf-α* level was reduced by 33%, 37%, and 60% (*p* = 0.028) in the MF-5, MF-25, and MF-125 groups, respectively. *Il-10* level was reduced by 36%, 62% (*p* = 0.016), and 9% when compared to the MCD group after FENO treatment (Figures 4C, D), suggesting the anti-inflammatory effects of FENO (25 mg/kg·BID) was strongest among the 3 dose groups.

In the preventive CDAHFD model, the levels of *Ccl2*, *Cxcl2*, *Tnf-α*, and *Il-10* were increased 35-, 7-, 16-, and 7-fold, respectively, when compared to the CDAHFD-CON group (*p* < 0.001). In the CF-25 group, the above mRNA levels were significantly reduced after being treated with the regime of FENO (25 mg/kg·BID), which was also the strongest among the 3 dose levels. The difference was statistically significant in comparison to the CDAHFD group (*p* < 0.001, Figures 4E–H).

**FIGURE 5:**
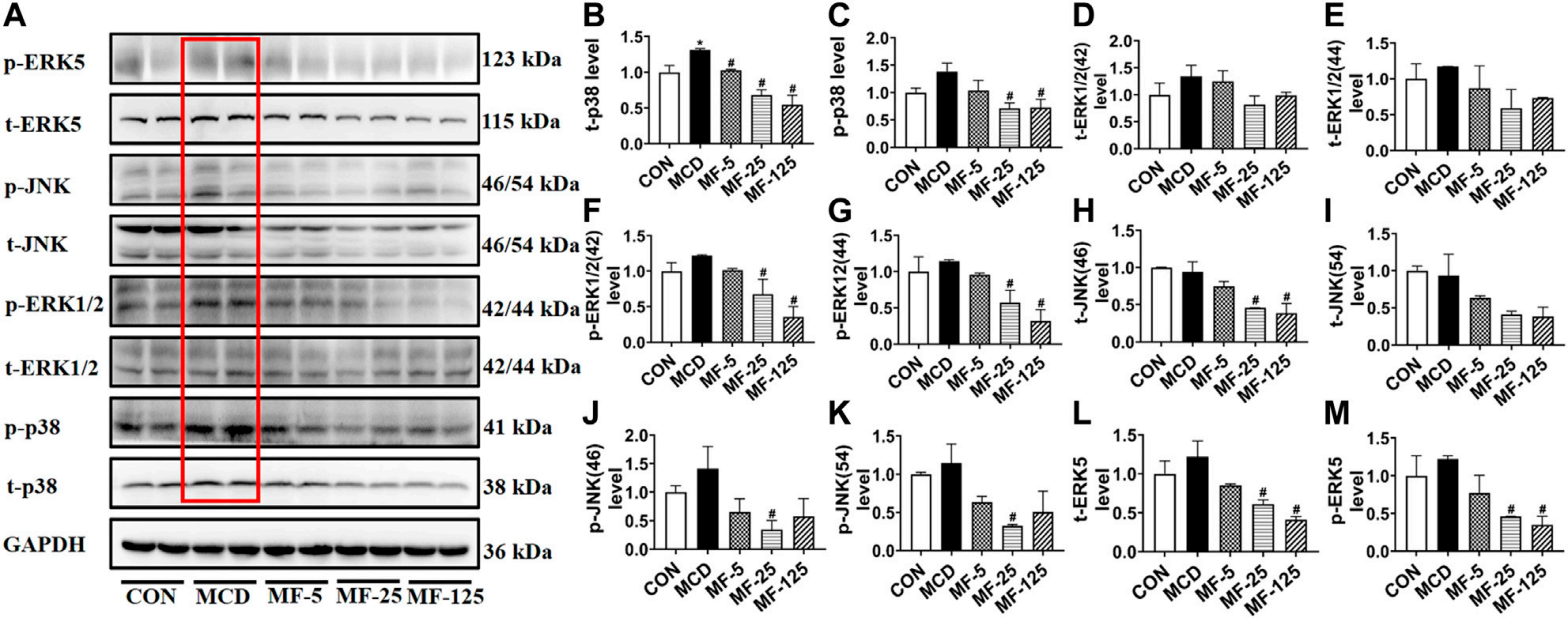
FENO’s therapeutic effects for the MCD model involved inhibition of the MAPK pathways. **(A)** Western blots of components of the MAPK pathways in liver extracts of MCD mice. **(B–M)** The quantification of MAPK pathways families include p38, ERK1/2, JNK and ERK5. GAPDH is used for loading control. (*n* = 3 for each group).

**FIGURE 6:**
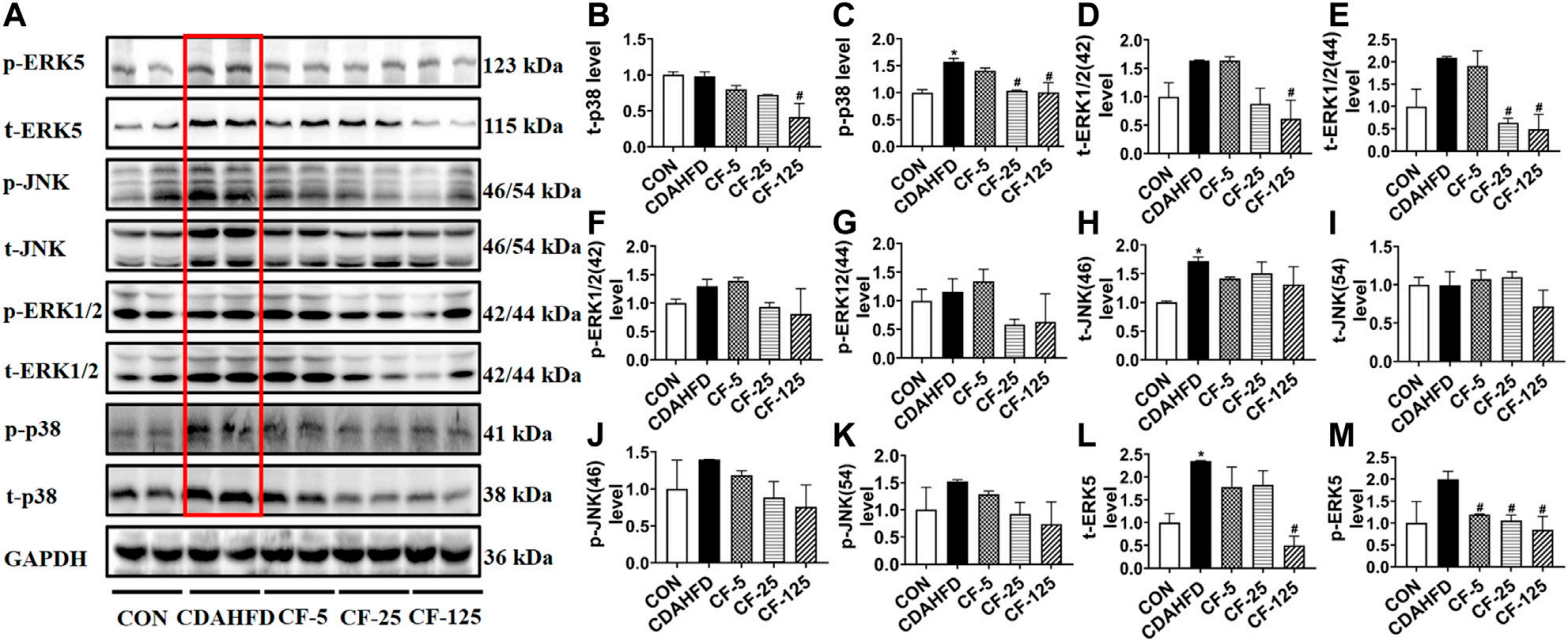
FENO’s preventive effects for the CDAHFD model involved inhibition of the MAPK pathways. **(A)** Western blots of components of the MAPK pathways in liver extracts of CDAHFD mice. **(B–M)** The quantification of MAPK pathways families include p38, ERK1/2, JNK and ERK5. GAPDH is used for loading control. (*n* = 3 for each group).

To investigate the anti-inflammation mechanism of FENO on NASH, the four typical MAPK pathways including p38, ERK1/2, JNK and ERK5 were measured (Figure 5A). Western Blots showed that the protein expression levels of phosphorylated p38, ERK1/2, JNK and ERK5 were upregulated in the therapeutic MCD model. The phosphorylation of MAPK pathways was downregulated after FENO treatment in the MCD model (Figures 5B–M). In the preventive CDAHFD model, the protein expression levels of phosphorylated p38, JNK and ERK5 were upregulated (Figure 6A). The phosphorylation of MAPK pathways was in downregulated after FENO treatment in the CDAHFD model (Figures 6B–M). Particularly at FENO 25 and 125 mg/kg·BID dosages, FENO therapy decreased the expression of phosphorylated proteins in both NASH models. These results indicated that the expression of inflammatory proteins and MAPK pathway activities were downregulated after FENO treatment. The difference between FENO 25 and 125 mg/kg·BID, however, was not appreciably different. FENO (25 mg/kg·BID) was therefore adequate to attenuate the inflammatory response in NASH.

**FIGURE 7:**
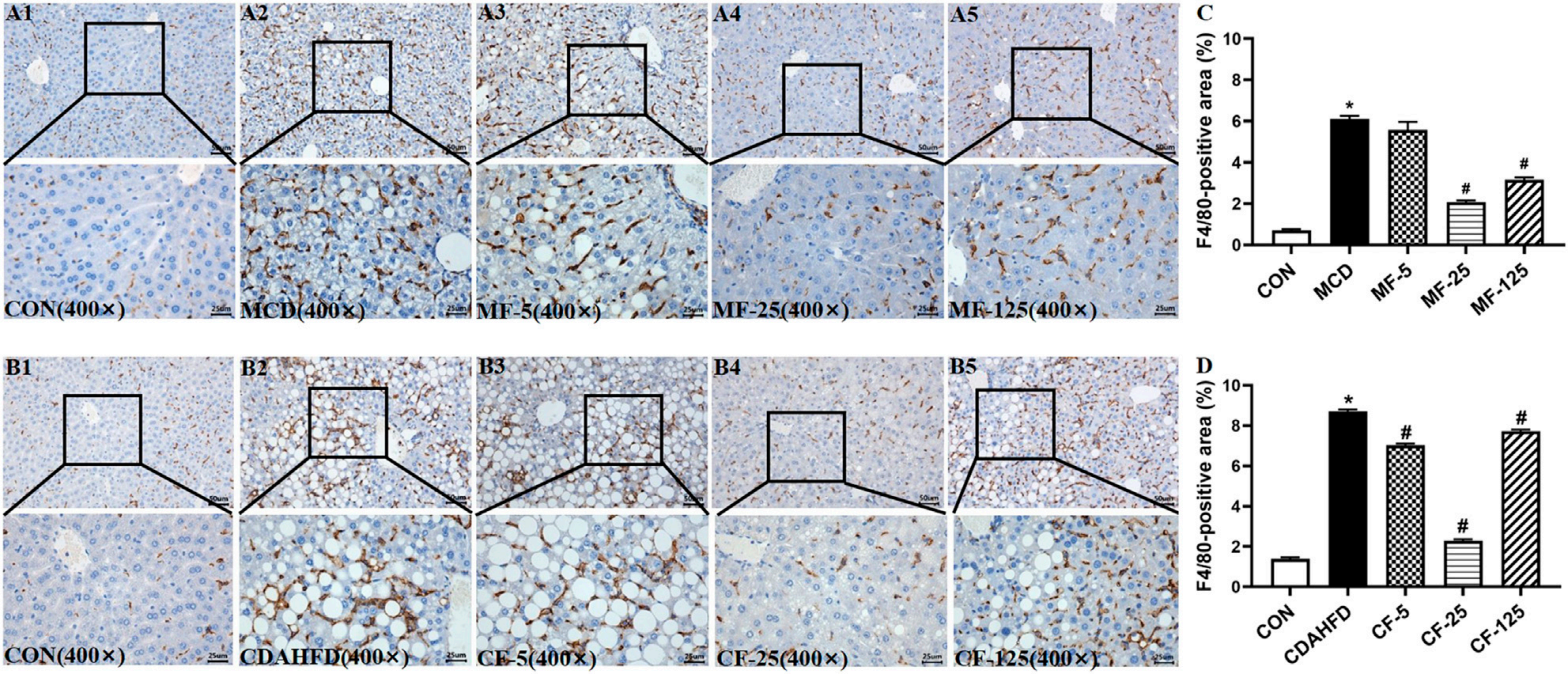
Macrophage number was decreased most by the regime of FENO 25 mg/kg·BID. Bar = 50 μm and 25 μm Representative immunohistochemical staining of F4/80 in the livers of the MCD model **(A1–A5)** and the CDAHFD model **(B1–B5)**. **(C)** F4/80-positive area of the MCD model. **(D)** F4/80-positive area of the CDAHFD model.

Kupffer cells were a group of macrophages in the liver tissues. They were specialized macrophages that were part of the mononuclear phagocyte system (Dixon et al., 2013). Kupffer cells responded to liver injury by releasing a range of inflammatory mediators, which contributed to inducing an inflammatory process (Tsutsui and Nishiguchi, 2014). F4/80 was an antigen marker for liver mature macrophage in mice (Lee et al., 1985). F4/80 immunohistochemical staining demonstrated that the hepatic infiltration of macrophages increased in the liver of two NASH models, whereas FENO (25 mg/kg·BID) treatment significantly reduced the number of macrophages (Figure 7). These data suggested that the anti-inflammation ability of FENO (25 mg/kg/·BID) was the highest among the 3 dose groups.

### 3.4 FENO 25 mg/kg·BID caused the least side effects in two NASH models

**FIGURE 8:**
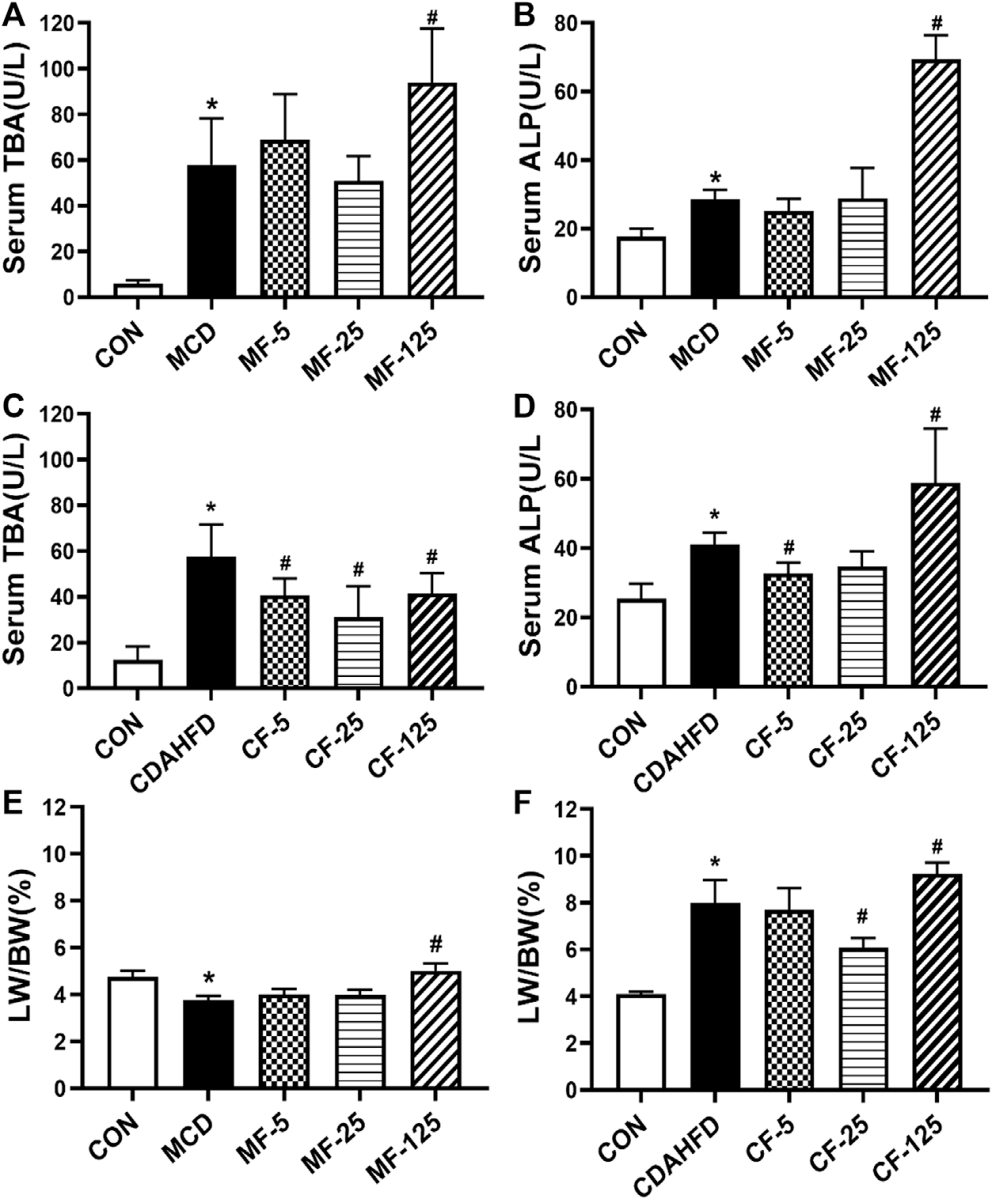
Toxic response of FENO 125 mg/kg·BID in two NASH models. **(A, B)** TBA and ALP in serum in the MCD model. **(C, D)** TBA and ALP in serum in the CDAHFD model. **(E, F)** The liver to body weight ratio of two mouse lines, respectively. The data were expressed as mean ± SD (*n* = 5, *p < 0.05, compared with CON group; ^#^ *p*< 0.05, compared with MCD or CDAHFD group).

In the therapeutic MCD model, the serum levels of TBA and ALP in the MCD group were 10- and 2-fold higher than those in the MCD-CON group (*p* = 0.005 and *p* = 0.034), suggesting that the mice developed hepatic cholestasis. Serum ALP in the MF-5 group treated with FENO showed slight improvement. In the MF-125 group, serum TBA and ALP were 2-fold higher than those in the MCD group (*p* = 0.031 and *p* < 0.001, Figures 8A, B). This result indicated FENO (125 mg/kg·BID) caused increase of bile acid load.

In the preventive CDAHFD model, the levels of TBA and ALP in the CDAHFD group were 5- and 2-fold higher than in the CDAHFD-CON group (*p* < 0.001 and *p* = 0.019). In the CF-25 group, serum TBA levels dropped by 46% (*p* < 0.001). Importantly, serum ALP decreased by 20% and 15% in the CF-5 and CF-25 groups, but increased by 30% in the CF-125 group (*p* < 0.001, Figures 8C, D). The above results showed FENO (125 mg/kg·BID) also caused increase of bile acid load. In contrast, FENO (25 mg/kg·BID) was safer in term of bile acid metabolism.

In terms of hepatomegaly, the MCD diet reduced the liver index by 21% in the MCD-group (*p* = 0.041). Compared to the MCD group, the liver index of the MF-5 and MF-25 groups did not change significantly, but the liver index of the MF-125 group doubled (*p* < 0.001, Figure 8E). In the CDAHFD group, the liver index was 2-fold higher than in the CDAHFD-CON group (*p* < 0.001). CF-5, CF-25, and CF-125 dose groups all increased the liver index. Following FENO treatment, the liver index was decreased by 4% and 24% (*p* < 0.001) in the CF-5 and CF-25 groups, respectively, but was elevated 13% in the CF-125 group compared to the CDAHFD group (*p* = 0.008, Figure 8F). The indicators of hepatic cholestasis and the liver index indicating that (25 mg/kg·BID) FENO was safer and (125 mg/kg·BID) FENO may might cause drug-induced intrahepatic cholestasis and even the potential of hepatocyte proliferation. In the preventive CDAHFD model, the liver index was lower in the CF-25 group compared to the CF-125 group, demonstrating that the proliferative effect of FENO (25 mg/kg·BID) was compromised by the drug’s ability to reduce hepatic lipid accumulation.

**FIGURE 9:**
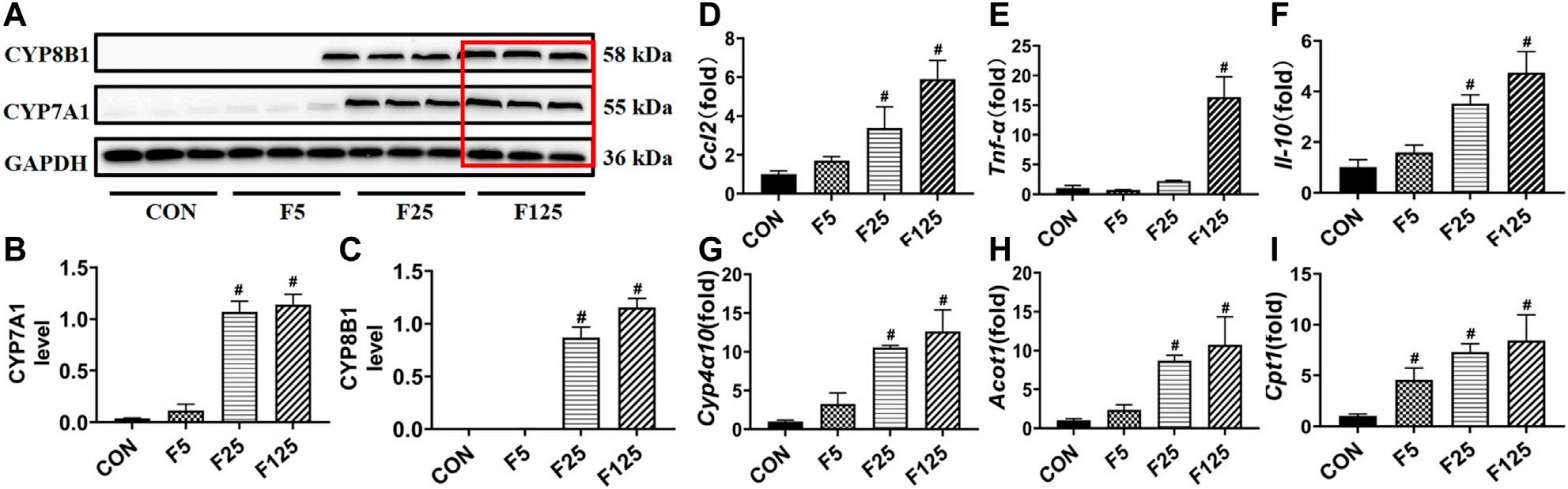
Toxic risk not the lipid catabolism of FENO 25 mg/kg·BID decreased compared with FENO 125 mg/kg·BID. **(A)** WB analysis of bile acid synthesis genes, CYP7A1 and CYP8B1; **(B, C)** The quantification of CYP7A1 and CYP8B1. **(D–F)** Hepatic expression of genes related to inflammation; **(G–I)** Hepatic expression of genes involved in PPARα mediated lipid metabolism. The mRNA levels were measured by quantitative PCR and normalized by *18S rRNA*. The data were expressed as mean ± SD (*n* = 5, *p < 0.05, compared with CON group; ^#^ *p*< 0.05, compared with MCD or CDAHFD group).

**FIGURE 10:**
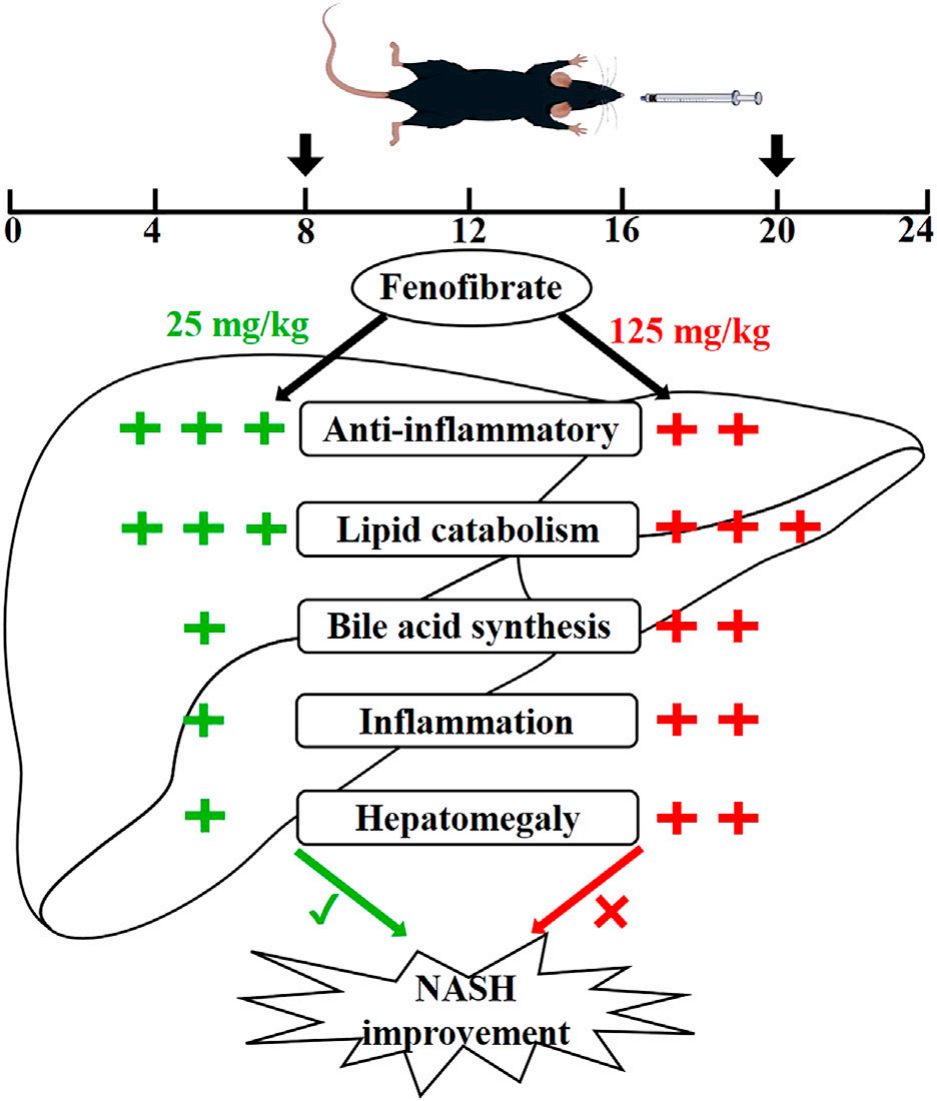
Proposed superior action mode of FENO 25 mg/kg·BID versus FENO 125 mg/kg·BID. In two NASH models, mice were treated with FENO by oral gavage twice a day (8 a.m. and 8 p.m.). FENO (25 mg/kg·BID) showed the best anti-inflammation effects compared with FENO (125 mg/kg·BID), and both had comparable lipid catabolic effects. However, FENO (125 mg/kg·BID) increased bile acid synthesis, triggered inflammation and induced the hepatomegaly. (‘+‘: the degree of action; ‘√‘: the best effect; ‘×‘: less effective.)

In the third experiment, the actions of FENO on bile acid synthesis, lipid metabolism, and inflammation response were investigated using normal C57BL/6 mice. In Western Blot analysis, the expression of bile acid synthesizing enzymes CYP7A1 and CYP8B1, were obviously increased in F125 group (Figures 9A–C, Figure 10). This could explain why FENO (125 mg/kg·BID) caused the increase in bile acid load. For the toxicity risk evaluation, ALT/AST were measured and HE staining was performed. They were not modified by 3 dose levels of FENO (data not shown). Accordingly, more up-stream and sensitive markers *Ccl2*, *Tnf-α* and *Il-10* were measured. In Q-PCR analysis, the expression of inflammatory chemokines, pro-inflammatory cytokines and protective inflammatory cytokines were significantly increased in the F125 group (*p*< 0.001, Figures 9D–F), suggesting (125 mg/kg·BID) FENO triggered inflammation response. Besides, PPARα-mediated lipid catabolism genes, *CPY4a10*, *Acot1* and *Cpt1*, were elevated, but their increases were quite similar between FENO 25 and FENO 125 mg/kg·BID regimes (Figures 9G–I). The findings demonstrated that FENO (25 mg/kg·BID) treated two NASH models through both anti-inflammation and lipid catabolism. And there was no discernible difference between the F25 and F125 groups in the lipid catabolism.

## 4 Discussion

The MCD diet was a widely used feeding regime to induce NASH, which can quickly duplicate the NASH model. The body weight of mice was sharply reduced on the MCD diet (Mridha et al., 2017), suggesting it was not suitable for long-term studies. The results acquired in our study are consistent with the published data. The CDAHFD in this study, which consists of 45% fat and an additional 1% cholesterol more closely resemble the human dietary pattern. It can not only mimic human NASH with steatosis, inflammation, and fibrosis in a short period, but also overcome significant body weight loss (Shimozono et al., 2015). In our pilot study, the CDAHFD model exhibited excessive lipid accumulation and the inflammation surpassed the reversible therapeutic check-point (data not shown) within 1 week. Thus, the MCD diet was used as a therapeutic model in our study. FENO was administered preventively when CDAHFD was used to challenge the mice. In this design, the results could be relevant to the treatment of chronic liver diseases.

Studies showed that FENO, a hypolipidemic drug, showed a good anti-inflammatory effect in addition to its therapeutic action of managing lipid metabolism. In clinical trials, FENO (300 mg/day) improved insulin resistance, cirrhosis, and fibrosis in NAFLD (El-Haggar and Mostafa, 2015). Animal models also indicated that FENO reduced hepatic steatosis and had anti-inflammatory and anti-fibrotic abilities (van der Veen et al., 2017; Lefere et al., 2020). However, several investigations reported conflicting results. In NAFLD patients, (200 mg/day) FNEO improved blood glucose levels and decreased the prevalence of metabolic syndrome without significantly improving liver histology (Fernández-Miranda et al., 2008). In NASH mice administered 500 mg/kg·day of FENO, the liver TG increased dramatically, and hepatic steatosis was apparent (Yan et al., 2014). As a result, current clinical and animal findings regarding the effect of FENO on NASH treatment are contradictory. The therapeutic effect of low-dose FENO was also observed in different diseases. In diabetic rats, FENO (30 mg/kg·day) showed a protective action on diabetic nephropathy (Kadian et al., 2013). FENO (50 mg/kg·day) was reported to effectively reverse alpha-naphthylisothiocyanate-induced liver injury in mice by inhibiting the JNK inflammatory pathway, whereas the lower dose and the higher dose was ineffective, indicating that the anti-inflammatory effect of FENO treatment was strictly dose-dependent (Dai et al., 2017; Dai et al., 2020). These new therapeutic actions of low-dose FENO against metabolic disease suggest its potential usage in dealing with NAFLD.

The above low dose dependence of FENO therapy was remarkable and deserved investigation. Pharmacokinetically, FENO had a half-life of approximately 20 h in humans and 6.8 h in mice (Bijland et al., 2010; Li et al., 2019). Besides, both the dosing protocol in earlier animal studies, as well as the clinical administration of FENO, were once daily. Considering the short half-life and the activation of FENO on the inflammatory response, twice-daily dosing may be more acceptable in rodent models, which may be the primary cause of the contradictory reports mentioned above. Thus, our study used a pharmacokinetic-based protocol of a twice-daily regime.

In our study, as a therapeutic model, the MCD model showed a significant body weight reduction. The CDAHFD model, as a preventive treatment model, showed a steady body weight change. There was no appreciable difference in body weight in two models following 3 doses of FENO treatment. And there was a significant decrease in serum ALT, AST and inflammatory factors levels in term of liver injury and inflammation. Histopathologically, the MCD and CDAHFD models exhibited steatosis and inflammatory cell infiltration, with the greatest improvement in NASH occurring at 25 mg/kg·BID. However, the CDAHFD model showed no fibrotic modifications, which may be due to the short time of modeling and no fibrosis development. In both therapeutic and preventive treatment models, FENO (125 mg/kg·BID) led to higher serum TBA and ALP levels and liver inflammatory markers than FENO (25 mg/kg·BID). Experiment 3 therefore demonstrated the toxicity risk of FENO alone on bile acid metabolism, lipid metabolism, and inflammation markers.

PPARα is a nuclear receptor that, upon activation, increases expression of a multitude of genes involved in fatty acid oxidation (Kersten et al., 1999). In our study, the expression of genes involved in fatty acid oxidation (*Cyp4a-10, Acot1, Cpt1 and Ehhadh*) were activated after FENO treatment between MCD and CDAHFD models (data are not shown). However, there was no significant difference between 25 and 125 mg/kg. In terms of liver inflammation, ALT and AST are primarily produced by liver and cardiac tissues, particularly liver cells. When the liver cells and mitochondria were be injured, they would be released into the bloodstream, resulting in a significant rise in serum ALT and AST levels. The inflammatory factors *Ccl2, Cxcl2, Tnf-α and Il-10*, as upstream of inflammation, are more sensitive than ALT and AST. *Ccl2* and *Cxcl2* are chemokines that function in recruiting neutrophils. When tissues were challenged, chemokines mediated the occurrence and development of inflammatory responses (De Filippo et al., 2013; Zhang et al., 2017). *Tnf-α* is a pro-inflammatory cytokine that is predominantly produced by macrophages and monocytes. Evidence indicate that *Tnf-α* was associated with NAFLD (Loman et al., 2018). It can be elevated in diseases such as chronic inflammatory response, NAFLD/NASH, rheumatoid arthritis, and others (Zhang et al., 2015; Armaka et al., 2022). *Il-10* was a protective regulator of the inflammatory response, which means *Il-10* may inhibit T cells from secreting a variety of inflammatory factors, such as *Il-2*, *Il-6* and *Tnf-α* (Holan et al., 2014). In this study, *Il-10* was also elevated when other inflammatory factors increased. Based on the biochemical and pathological results, therapeutically and preventatively, FENO (25 mg/kg·BID) reduced lipid accumulation and also delivered anti-inflammatory benefits.

FENO also caused many risks in the clinic. In hyperlipidemic patients, FENO increased ALT and AST levels relating to liver function (Oikawa et al., 2017). Long-term FENO administration led to an abnormal increase in dichloroacetic acid in liver tissue, which might exacerbate cholelithiasis (Hassoun et al., 2010). In term of myotoxicity, FENO therapy increased the risk of myoglobinemia in a rat model (Pierno et al., 2006). Besides, prolonged administration of FENO caused hepatocarcinogenesis in rodents (Peters et al., 2005). In this study, the cholestatic biomarkers TBA and ALP in the MF-125 and CF125 groups were considerably elevated when compared to the CON group. The liver index of the MCD and CDAHFD groups were both increased by FENO (125 mg/kg·BID). Additionally, the third experiment was conducted for 5 days, which showed the toxic effects of FENO. The lipid catabolism and inflammatory factors (*Ccl2, Tnf-α, Il-10, Cyp4α10, Acot1 and Cpt1*) increased in a dose-dependent manner. These factors were used as sensitive indicators rather than phenotypic ones. The lipolytic effects of FENO (25 mg/kg) was comparable to that of 125 mg/kg. FENO at 125 mg/kg, however, was found to increase the bile acid synthesis and inflammatory response. Above all, although the adverse effects of FENO (5 mg/kg·BID) in this trial were minor, the therapeutic benefits were negligible. FENO (125 mg/kg·BID), on the other hand, triggered inflammatory response, an increase of bile acid load, as well as a proliferative response in the liver. Thus, FENO (25 mg/kg·BID) was the most efficient in alleviating NASH.

There were several limitations in our study. First of all, CDAHFD is a model with severe steatosis and inflammation, which exceeded the reversible therapeutic check point quickly. Thus, in our study, CDAHFD was only used as a preventative model. Secondly, translational medicine remained to be investigated following these animal experiments. Finally, the MAPK family includes four primary pathways. It remains to be investigated to explore which pathway mediated the anti-inflammatory effect and what the underlying molecular mechanisms were.

Conclusively in this study, FENO (25 mg/kg·BID) treatment mitigated NASH by inhibiting hepatic steatosis, inflammation and fibrosis in both therapeutic and preventive treatment models, which stemmed from its action on lipid catabolism and anti-inflammation. FENO (5 mg/kg·BID) showed little effect in hepatic steatosis and inflammation, neither the adverse effects. FENO (125 mg/kg·BID) aggravated liver inflammation, increased bile acid load, and promoted the potential of liver proliferation. Also, the toxic risk of high-dose FENO were confirmed in the normal mice model. Considering species differences in pharmacokinetics and the low risk of dose-dependent adverse reactions, translational medicine is warranted to prove the effectiveness of FENO (25 mg/kg·BID) in the clinic.

## Data Availability

The original contributions presented in the study are included in the article/Supplementary Materials, further inquiries can be directed to the corresponding authors.
