# Supplementary material for: Therapeutic effect of fenofibrate for non-alcoholic steatohepatitis in mouse models is dependent on regime design
Source: Front Pharmacol. 2023 May 12;14:1190458. doi: 10.3389/fphar.2023.1190458 (PMC10213340; doi:10.3389/fphar.2023.1190458)
Supplement: Supplementary file 1 [file Table1.DOCX]

Supplementary Table S1

| Genes | Forward | Reverse |
| --- | --- | --- |
| *Ccl2* | TTAAAAACCTGGATCGGAACCAA | GCATTAGCTTCAGATTTACGGGT |
| *Cxcl2* | CCAACCACCAGGCTACAGG | GCGTCACACTCAAGCTCTG |
| *Tnf-α* | AGGGTCTGGGCCATAGAACT | CCACCACGCTCTTCTGTCTAC |
| *Il-10* | TGTCAAATTCATTCATGGCCT | ATCGATTTCTCCCCTGTGAA |
| *Cyp4α10* | TTCCCTGATGGACGCTCTTAT | CGAAACCTGGAAGGGTCAAAC |
| *Acot1* | CCCCTGTGACTATCCTGAGAA | CAAACACTCACTACCCAACTGT |
| *Cpt1* | TGGCATCATCACTGGTGTGTT | GTCTAGGGTCCGATTGATCTTTC |
| *18 S* | ATTGGAGCTGGAATTACCGC | CGGCTACCACATCCAAGGAA |
